# Methodological considerations for validating a machine learning model predicting recurrence after ESD in superficial esophageal cancer

**DOI:** 10.1080/07853890.2026.2634569

**Published:** 2026-02-25

**Authors:** Dandan Song, Shufu Hou, Jing Gao

**Affiliations:** ^a^ Department of Neurology, Shandong Provincial Third Hospital, Shandong University, Jinan, China; ^b^ Department of Gastrointestinal Surgery, Central Hospital Affiliated to Shandong First Medical University, Jinan, China; ^c^ Department of Labor Union Office, Shandong Provincial Third Hospital, Shandong University, Jinan, China

We read with great interest the article by Zhu et al., which developed an interpretable Random Forest model to predict recurrence after endoscopic submucosal dissection (ESD) for superficial oesophageal squamous cell carcinoma (SESCC) [1]. The integration of biological age and systemic inflammatory markers, alongside robust temporal validation and SHAP-based interpretability, represents a commendable and clinically relevant contribution to personalized surveillance strategies.

However, we wish to raise two methodological points that are crucial for interpreting the study’s conclusions and ensuring its reproducibility.

First, the statistical independence between model development and validation appears compromised. The univariate/multivariate logistic regression and restricted cubic spline (RCS) analyses for identifying “independent risk factors” and dose-response relationships were performed on the entire cohort, which included the temporal validation set (2022–2023) [1]. In machine learning research, using the validation data in any part of the feature analysis or model inference stage can lead to optimistic performance estimates and undermine the validity of the reported associations [2]. To strengthen the work, we suggest the authors re-perform these foundational statistical analyses using only the development cohort (2016–2021). This would ensure a clean separation between the data used to understand variable relationships and the data used for final, unbiased evaluation.

Second, the choice of a binary outcome (recurrence yes/no) without accounting for variable follow-up time and censoring may introduce bias. Patients with shorter follow-up are inherently less likely to have a recorded recurrence, potentially conflating the risk of recurrence with the duration of observation. Given that metachronous recurrence is defined as occurring >12 months post-ESD, a time-to-event analysis (e.g. Cox regression or Random Survival Forest) would be more appropriate [3]. This framework directly handles censored data and provides hazard ratios, offering a more nuanced and clinically intuitive measure of risk over time. We encourage the authors to consider presenting a sensitivity analysis using survival modeling, which would significantly enhance the model’s robustness and clinical applicability [4].

Addressing these points would not only solidify the methodological rigor of this promising study but also increase confidence in its clinical translation.

## Data Availability

Data sharing is not applicable to this article as no data were created or analysed in this research.

## References

[1] Zhu Z, Li Q, Chen W, et al. Development and validation of an interpretable Random Forest model for predicting recurrence after endoscopic submucosal dissection in superficial oesophageal squamous cell carcinoma. Ann Med. 2026;58(1):2606571. doi: 10.1080/07853890.2025.2606571. 41439278 PMC12777826

[2] Yin X, Cui Y, Liu T, et al. Development and validation a radiomics combined clinical model predicts treatment response for esophageal squamous cell carcinoma patients. BMC Gastroenterol. 2025;25(1):313. doi: 10.1186/s12876-025-03899-8. 40301780 PMC12042612

[3] Wang Y, Wu Q. Development and validation of an interpretable ML model for survival prediction in unresectable ESCC with immunochemotherapy. Cancer Manag Res. 2025;17:2609–2619. doi: 10.2147/CMAR.S532944. 41209035 PMC12595924

[4] Zhou X, Wu B, Tang D, et al. Risk prediction models of esophageal strictures after endoscopic submucosal dissection: a systematic review and meta-analysis. Dig Dis Sci. 2025;70(12):4046–4059. doi: 10.1007/s10620-025-09217-2. 40715688

